# The effects of maternal separation on behaviours under social-housing environments in adult male C57BL/6 mice

**DOI:** 10.1038/s41598-020-80206-3

**Published:** 2021-01-12

**Authors:** Nozomi Endo, Manabu Makinodan, Takayo Mannari-Sasagawa, Noriko Horii-Hayashi, Nami Somayama, Takashi Komori, Toshifumi Kishimoto, Mayumi Nishi

**Affiliations:** 1 Department of Anatomy and Cell Biology, Nara Medical University, 840 Shijo-cho, Kashihara, Nara 634-8521 Japan; 2 Department of Psychiatry, Nara Medical University, Kashihara, Nara 634-8521 Japan; 3 Faculty of Human Life and Environment, Nara Women’s University, Nara, 630-8506 Japan

**Keywords:** Social behaviour, Psychiatric disorders

## Abstract

Adverse experience in early life can affect the formation of neuronal circuits during postnatal development and exert long-lasting influences on neural functions that can lead to the development of a variety of psychiatric disorders including depression, anxiety disorders, and post-traumatic stress disorder. Many studies have demonstrated that daily repeated maternal separation, an animal model of early-life stress, can induce impairments in emotional behaviours and cognitive function during adolescence and adulthood. However, the behavioural phenotypes of maternally separated mice under long-term group-housing conditions are largely unknown. In this study, we applied our newly developed assay system to investigate the effects of maternal separation on behaviours under group-housing conditions during four days of continuous observations. Using our system, we found that repeated maternal separation resulted in inappropriate social distance from cagemates, altered approach preferences to others, and induced a lower rank in the time spent on the running wheel under group-housing conditions in adult male mice. Focussing on these behavioural abnormalities that appear in an environment with a social context will be important insights to understand the pathogenesis of psychiatric disorders.

## Introduction

In this rapidly changing society, complexities in family dynamics as well as changes in other social environmental factors can greatly affect the mother–child relationship. Childhood abuse, including sexual contact, physical abuse, and severe neglect, is one of the highest-level risk factors for many neuropsychiatric disorders in adult patients^1–3^. Importantly, stressors occurring during critical periods of development, such as perinatal life, are known to produce adverse effects on numerous behaviours and physiological functions including growth, metabolism, reproduction, sleep, and inflammatory/immune response^4–7^. Therefore, early environmental insults or stressors likely cause a permanent, rather than a transient, effect on the organism. For example, interruptions of typical mother–pup interactions have been reported to induce persistent changes in the neurobiology, physiology, and emotional behaviour of adolescent and adult animals by disrupting the responsiveness of the hypothalamic–pituitary–adrenal (HPA) axis^8–11^. Many studies have shown that daily repeated maternal separation (MS) during the neonatal period, an animal model of early life stress, can induce subsequent mood and anxiety disorders during adulthood^12,13^. Neonatal MS also alters cognitive function in adulthood^14–17^. Recently, we showed that MS reduces reward-seeking behaviour in female mice assessed by a conditioned place preference test^18^. However, these conventional behavioural examinations using an MS mouse model were conducted under simplified artificial conditions for a short observational period. Moreover, social context plays a critical role in both the aetiology and expression of psychopathology in humans^19^. Analysis of animal behaviours in an environment with a social context is therefore essential to disentangling the neuronal circuitry underlying neurodevelopmental and neuropsychiatric disorders. Benner et al. (2014) reported that MS in male mice induces competitive subordinance in a group-housed environment using IntelliCage, a radio frequency identification (RFID)-based automated behaviour testing system^20^. Although IntelliCage can simultaneously analyse the behaviour of over a dozen group-housed RFID-tagged mice in the experimental cage for a long period of time, it is only capable of detecting when a mouse comes near the RFID reader located in the four corners of the cage; thus, it is unable to determine the location where the mouse was localized in the experimental cage. Consequently, the behavioural phenotypes of MS mice under group-housing conditions remain largely unknown.

In order to investigate mouse behaviour under group-housing conditions for a long period of time, we developed a behavioural analysis system, Multiple Animal Positioning System (MAPS), which can identify multiple mice in a group-housing environment and continuously localize each mouse in Cartesian coordinates over long durations. Using MAPS, we found that mice subjected to social isolation after weaning exhibited longer social distance and needed more time until huddle with cagemates than control mice^21^. We also showed that BTBR mice, a rodent model of autism spectrum disorder, exhibited lower activity levels in the dark phase and altered social behaviour compared to C57BL/6J mice, by using MAPS^22^. Importantly, the results of locomotor activity in the BTBR obtained by MAPS are different from those observed by using conventional open-field tests, which showed higher levels of locomotor activity in the BTBR mice^23–26^. Moreover, we recently made further improvements to the MAPS in order to the higher image acquisition rate from 1 frame per second (fps) to 10 fps. The purpose of this study was to clarify the behavioural phenotypes of MS mice under group-housing conditions using our assay system.

## Results

### MS mice display normal locomotor activity under group-housing conditions

**Figure 1:**
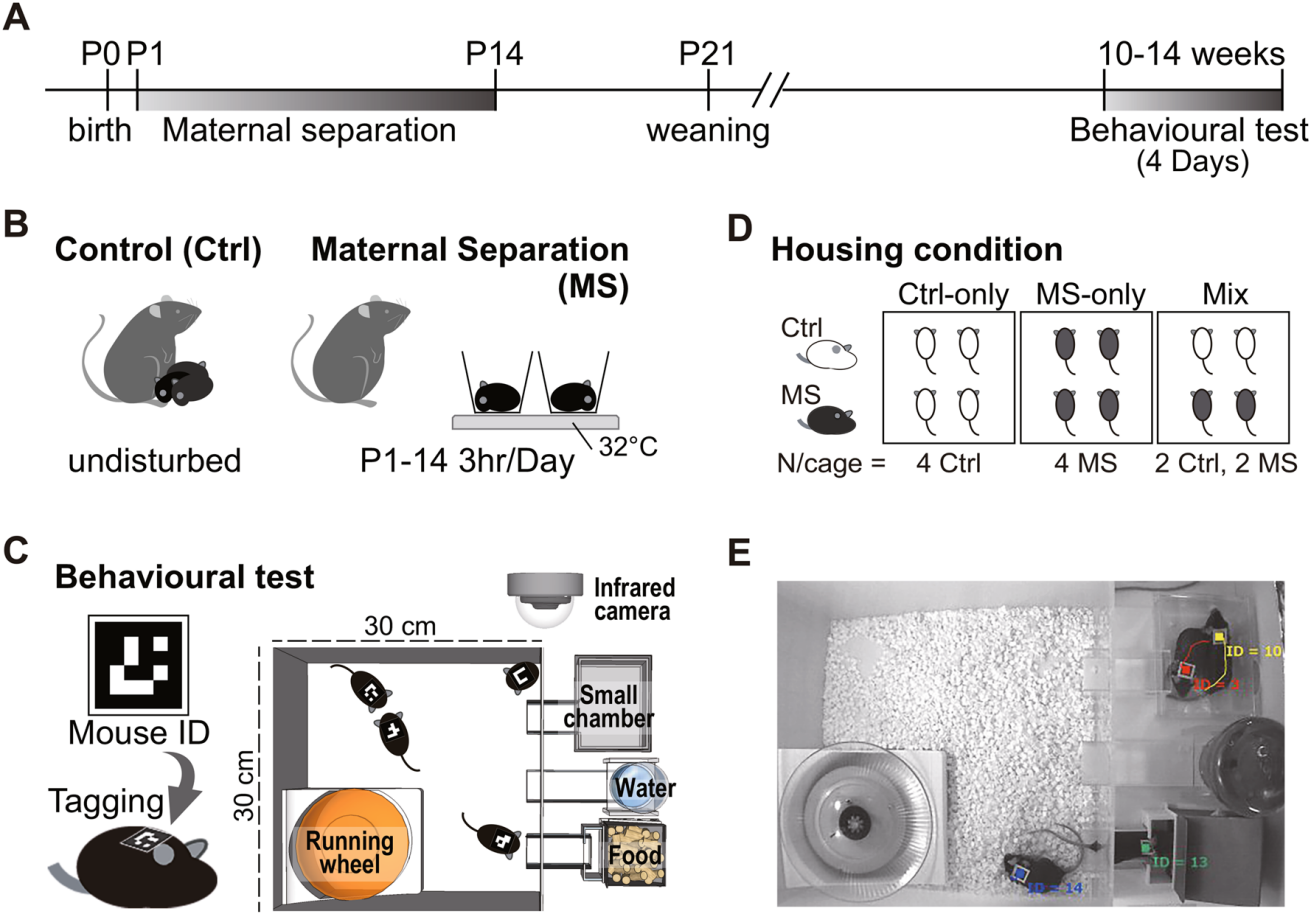
Summary of experimental design. (**A**) Schedule for maternal separation and behavioural analysis. (**B**) An illustration for the maternal separation paradigm. (**C**) Schematic of behavioural analysis under group-housing conditions. Each mouse was tagged with a mouse ID on its back. Four adult male mice that had not previously been exposed to one another were placed in an experimental cage. (**D**) An illustration for the combination of 4 mice for behavioural analysis (housing condition). (**E**) A representative still image in which each mouse ID was detected by our system.

**Figure 2:**
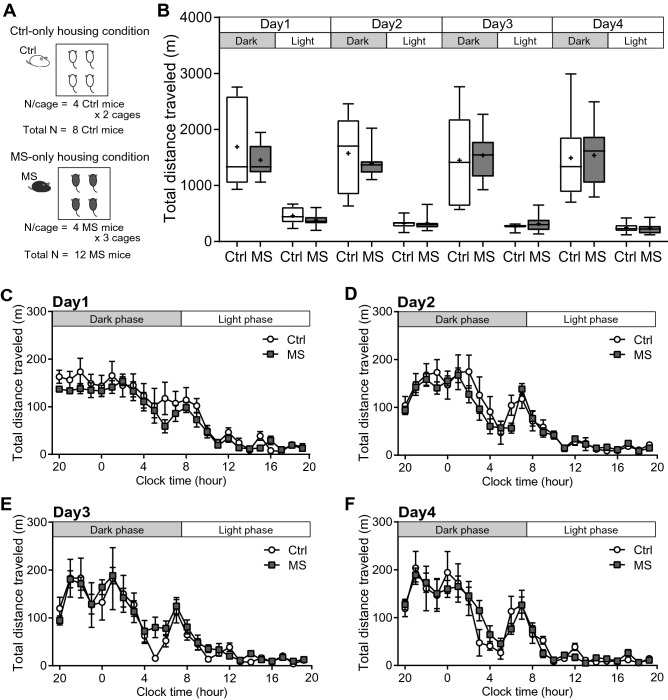
Locomotor activity under the Ctrl-only and MS-only housing conditions. (**A**) Experimental design for the Ctrl-only and MS-only housing conditions. Four Ctrl or four MS mice (all previously unexposed to one another) were housed per cage. Two cages were tested in the Ctrl-only housing condition and three cages in the MS-only housing condition (Ctrl: total n = 8; MS: total n = 12). (**B**) The total distance travelled during the dark and light phases (12-h epochs) on Days 1–4. Centre lines show the medians; box limits indicate the 25th and 75th percentiles; the whiskers show maximum and minimum values; + show the means. Three-way ANOVA (Group × Day × Dark/Light phase). (**C–F**) The total distance travelled divided into 1 h bins on Days 1–4. Data are shown as the mean ± SE. Three-way ANOVA (Group × Day × Hour). For ease of viewing, the graphs were presented separately for each day.

To examine how early life experience affects behaviours in adulthood under group-housing conditions, we assessed behavioural outcomes in MS and control (Ctrl) mice for four consecutive days (Fig. 1). Four adult male mice that had not previously been exposed to one another were introduced into one of the experimental cages (Supplementary Video S1). We first assessed locomotor activity in four Ctrl mice (Ctrl-only) and four MS mice (MS-only) (Fig. 2A). The locomotor activity of the MS mice during the dark and light phases was comparable to that of the Ctrl mice on Days 1–4 (f_(1, 54)_ = 0.10, *p* = 0.75; Fig. 2B). Furthermore, we took 1 h bins of locomotor activity data to visualize the circadian rhythms and found that there was no significant difference between these two groups (f_(1, 1242)_ = 0.10, *p* = 0.75; Fig. 2C–F). These results suggest that MS has no effect on locomotor activity or circadian rhythms under group-housing condition. In addition, we confirmed the effect of MS on locomotor activity in the conventional open-field test. The open-field test results showed that there was no significant difference in the total distance travelled, the time spent in the centre and the number of entries to the centre, between groups (t = 1.28 df = 12.52, *p* = 0.23 in the total distance travelled, t = 1.70 df = 8.36, *p* = 0.13 in the time spent in centre, t = 1.07 df = 9.62, *p* = 0.31 in the number of entries to centre; Supplementary Fig. S1).

### MS treatment leads to inappropriate social distance from cagemates

**Figure 3:**
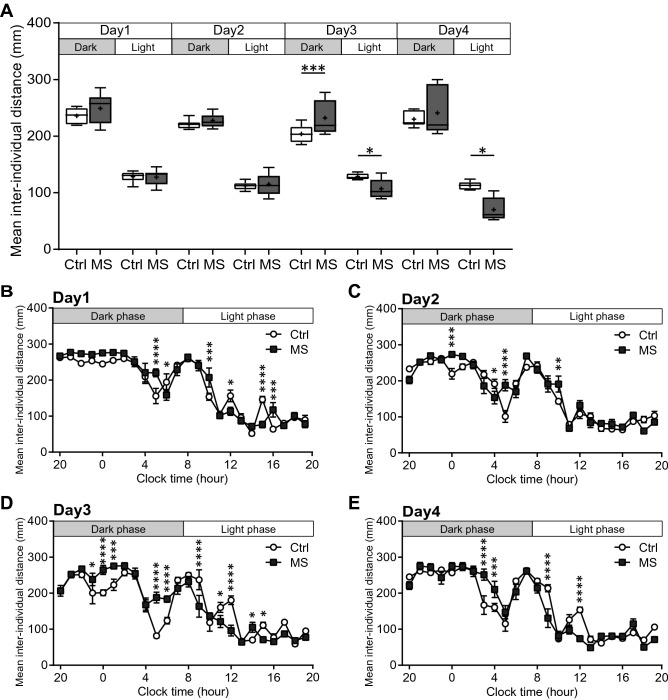
The mean inter-individual distances against the other three cagemates under the Ctrl-only and MS-only housing conditions. (**A**) The mean inter-individual distances against the other three cagemates during the dark and light phases (12-h epochs) on Days 1–4. Centre lines show the medians; box limits indicate the 25th and 75th percentiles; the whiskers show maximum and minimum values; + show the means. Data were analysed using three-way ANOVA (Group × Day × Dark/Light phase). As there was a significant interaction between factors (*p* = 0.008), a simple main effect test was performed. (**B–E**) The mean inter-individual distances against the other three cagemates divided into 1 h bins on Days 1–4. Data are shown as the mean ± SE. Data were analysed using three-way ANOVA (Group × Day × Hour). As there was a significant interaction between factors (*p* < 0.001), a simple main effect test was performed. For ease of viewing, the graphs were presented separately for each day. *p < 0.05, **p < 0.01, ***p < 0.005, ****p < 0.001. (Ctrl; n = 8, MS; n = 12).

We quantified the mean inter-individual distances against the other three cagemates under the Ctrl-only and MS-only housing conditions. Three-way ANOVA (Group × Day × Dark/Light phase) showed that there was a significant interaction between factors (*p* = 0.008), allowing for a simple main effect test to be performed. Here, we found that MS mice showed significantly longer distances than Ctrl mice on Day 3 in the dark period and significantly shorter distances than Ctrl mice on Days 3 and 4 in the light period (f_(1, 144)_ = 10.07, *p* = 0.0018 in the dark phase on Day 3, f_(1, 144)_ = 5.81, *p* = 0.017 in the light phase on Day 3, f_(1, 144)_ = 4.64, *p* = 0.033 in the light phase on Day 4; Fig. 3A). Furthermore, when data on the mean inter-individual distances was divided into 1 h bins, the time periods had a significant difference between groups on Day 1 and Day 2, but there was no consistent trend (MS mice showed longer or shorter distances in the time period, which was mixed). In contrast, on Day 3 and Day 4, the trends of the time periods were significantly different between the mouse groups, where there were longer distances in the dark period and shorter distances in the light period among MS mice compared with Ctrl mice (Fig. 3B–E).

**Figure 4:**
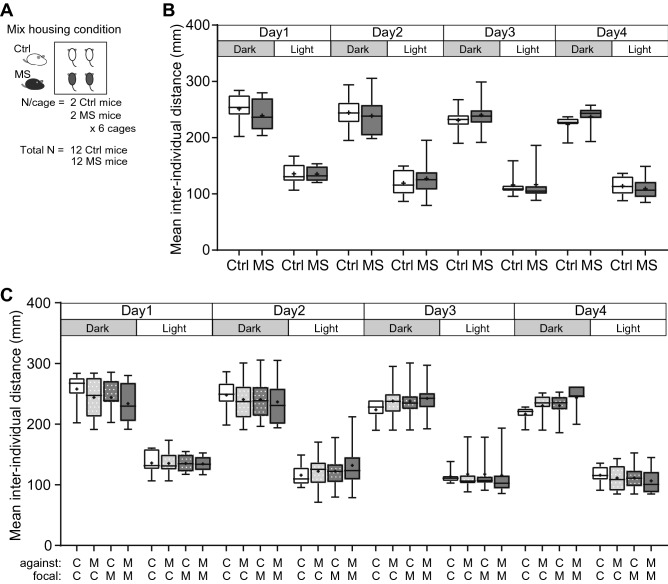
The mean inter-individual distances against the other three cagemates under the Mix housing conditions. (**A**) Experimental design for the Mix housing conditions. Two Ctrl or two MS mice (all previously unexposed to one another) were housed per cage. Six cages were tested in the Mix housing condition (Ctrl: total n = 12; MS: total n = 12). (**B**) The mean inter-individual distances against the other three cagemates during the dark and light phases (12-h epochs) on Days 1–4. (**C**) The mean inter-individual distances against the other Ctrl mouse and MS mice within a cage during the dark and light phases (12-h epochs) on Days 1–4. Centre lines show the medians; box limits indicate the 25th and 75th percentiles; the whiskers show maximum and minimum values; + show the means. Data were analysed using three-way ANOVA (Group × Day × Dark/Light phase). C means Ctrl, M means MS.

To examine whether the social distance of MS mice was affected by the makeup of their cagemates, we examined behaviours in a mixed housing condition (Mix), in which two Ctrl and two MS mice were placed in the same experimental cage (Fig. 4A). Three-way ANOVA (Group × Day × Dark/Light phase) showed that there was no significant differences in the mean inter-individual distances against the other three cagemates between mouse groups in the Mix housing condition (f_(1, 66)_ = 0.040, *p* = 0.84, Fig. 4B). Furthermore, we determined whether the social distance was influenced by the group identity of the partner mouse (Ctrl or MS mouse) by calculating the mean distances between individuals for each group of mouse pairs (focal-partner: Ctrl-Ctrl, Ctrl-MS, MS-Ctrl, MS–MS). Three-way ANOVA (Mice pair × Day × Dark/Light phase) showed that there was a significant interaction between factors (*p* = 0.038), allowing for a simple main effect test to be performed. We found that there was no difference in the mean inter-individual distances, regardless of whether the partner mouse was the Ctrl or MS group on any of the Days (Fig. 4C). Analysis of 1 h bin of inter-individual distances showed no significant differences between the mouse groups, either on average against the other three individuals or on average against each group of mice (Supplementary Figs. S2, S3). Thus, the inappropriate social distance among MS mice that was found in the Ctrl-only and MS-only housing conditions (Fig. 3) was not apparent in the Mix housing condition.

### Approach behaviour of MS mice was altered

**Figure 5:**
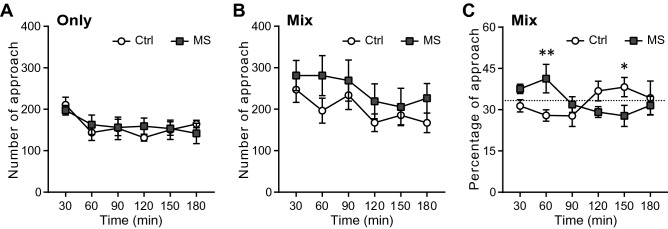
Approach behaviour in the novel environment. (**A**) The number of approaches under Ctrl-only and MS-only housing conditions. (Ctrl; n = 8, MS; n = 12) (**B**) The number of approaches under the Mix housing condition. (Ctrl; n = 12, MS; n = 12) (**C**) The percentage of approaches under the Mix housing condition. Data are shown as mean ± SE and were analysed using a two-way ANOVA (Group × Time). When a significant interaction between factors, a simple main effect test was performed. *p < 0.05, **p < 0.01. Dash lines indicate chance level. (Ctrl; n = 12, MS; n = 12).

Next, we quantified the approach behaviours to cagemates as another component of the social behavioural indices. We first focused on the first 3 h after the start of the experiment as a novel environment. In the novel environment, the number of total approaches of Ctrl mice and MS mice was not significantly different in any of the housing conditions (f_(1, 90)_ = 0.014, *p* = 0.91 in the Ctrl-only and MS-only housing conditions, f_(1, 110)_ = 1.196, *p* = 0.29 in the Mix housing condition; Fig. 5A-B). When data of the number of approaches on Days 1–4 were divided into light and dark periods or the 1 h bin, there were no significant differences between the groups of mice in any of the housing conditions (Supplementary Figs. S4–S5). Interestingly, the graph showing the percentage of approaches to the same group as themselves was drawn in a characteristic figure-eight shape in the novel environment in the Mix housing condition (Fig. 5C). Two-way ANOVA (Group × Time) result showed that there was a significant interaction between factors (*p* = 0.0033); therefore, a simple main effect test was performed. We found that there was no difference in the percentage of approaches during the first 30 min of the experiment (f_(1, 132)_ = 1.48, *p* = 0.23), while at 60 min the percentage of MS mice was significantly higher than that of Ctrl (f_(1, 132)_ = 6.96, *p* = 0.0094). The difference in the percentage of approaches remained non-significant between the groups until after 150 min when the percentage of MS mice became significantly lower than that of the Ctrl mice (f_(1, 132)_ = 4.25, *p* = 0.041), with no detectable differences at 180 min after the start of the experiment (f_(1, 132)_ = 0.29, *p* = 0.59). Although the percentage of approaches to the same group as themselves was not significantly different between mouse groups during the light and dark phase on Days 1–4 (f_(1, 66)_ = 0.46, p = 0.50; Supplementary Fig. S6A), there were time periods with a significant difference between mouse groups when the data on the percentage of approaches were divided into 1 h bins (Supplementary Fig. S6B–E). In particular, MS mice showed a tendency of occurring the time periods with lower percentage of approaches to the same group as themselves in the dark phase and higher percentages in the light phases on Day 3 and Day 4, compared with Ctrl mice.

### The duration of time spent on the running wheel

**Figure 6:**
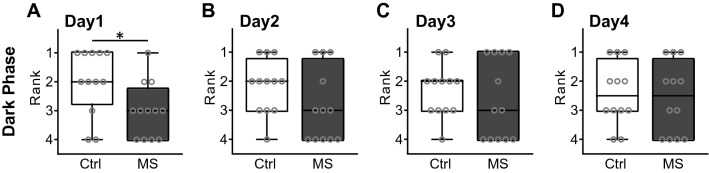
The rank of the duration of time on a running wheel. (**A–D**) The rank of the duration of time on a running wheel on Days 1–4 under the Mix housing condition. Centre lines show the medians; box limits indicate the 25th and 75th percentiles; the whiskers show maximum and minimum values. Data were analysed using the Mann–Whitney U Test. *p < 0.05. (Ctrl; n = 12, MS; n = 12).

Mice innately prefer voluntary wheel running. Finally, we quantified the duration of time spent on the running wheel during the dark phases on Days 1–4 and ranked according to the length of time spent on the running wheel. MS mice ranked significantly lower than Ctrl mice during the dark phase on Day 1 under the Mix housing condition (*p* = 0.047; Fig. 6).

### Basal corticosterone level in MS mice

We measured a basal plasma corticosterone level. MS mice showed significantly higher corticosterone levels than Ctrl mice (*p* = 0.025; Supplementary Fig. S7).

## Discussion

The present findings highlight the importance of investigating behavioural phenotypes of MS mice under long-term group-housing conditions. For the first time, we analysed various behaviours of adult male C57BL/6N mice exposed to perinatal repeated MS by using our assay system, an improved version of MAPS. We found several interesting behavioural phenotypes, including inappropriate social distance from cagemates, alternation in approach behaviour and disturbance of the lower rank in the time spent on the running wheel, which have never been observed by conventional behavioural analyses. To the best of our knowledge, these are the first behavioural phenotypes of MS mice to be found using their individual location data under group-housing conditions through day and night.

Previous studies that investigate the effect of maternal separation on locomotor activity using an open field test have generated inconsistent results. For example, there are reports that MS increased locomotor activity in rats^27^, MS reduced locomotor activity in mice^28^, and MS has no effect on locomotor activity in rats^29,30^ and mice^31,32^. Furthermore, Daniel Wang et al.^33^ published a systemic review and meta-analysis, examining the effects of MS on exploratory-defensive behaviour in mice and rats using the open field test and an elevated plus maze. They reported that MS was associated with increased defensive behaviour in rats. In contrast, MS did not alter exploratory behaviour in mice. In the present study, we found that locomotor activity is not affected by MS both in the open field test and under group housing condition. Furthermore, we showed that MS has no effect on the circadian rhythm of activity by analysing it for four consecutive days.

Social proximity, defined as the distance between individuals during social interactions, depends on the social relationships between individuals, not only in humans, but also in animals^34–37^. MS mice showed significantly longer distances than Ctrl mice on Days 3 in the Dark period and significantly shorter distances than Ctrl mice on Days 3 and 4 in the light period, under the Ctrl-only and MS-only housing conditions. As a general interpretation of social behaviour of the mouse, a higher number of approaches and/or longer time of contact with other mice is indicative of higher sociability. However, we think extreme contact with other mice may be related to other factors, such as weak vigilance or excessive affiliative behaviour, and have concerns about simply interpreting more contact as more sociability in rodents. Schultheiss and Brunstein^38^ argued about the duality of affiliative motivation in humans and suggested that there is also a dark side to affiliative motivation; it could be associated more with a fear of being alone and/or rejected than with the pleasure of being with others. Furthermore, considering the inter-individual distance from a personal space perspective in humans, an intrusion of personal space by others causes discomfort^39^. There have been some reports of patients with autism spectrum disorder that have a tendency to intrude into the space of others^40^ as well as reports of reduced personal space in individuals with autism spectrum disorder^41^. We are not sure yet whether mice have such a duality of affiliative behaviour or a concept of personal space. However, it is difficult to examine such affiliative behaviour in conventional, short-term tests in novel environments (i.e., reciprocal social interaction test or three-chamber test), which have been used for analysis in mouse models of psychiatric disorders. Further examinations using a long-term behavioural analysis under group-housing environments, such as in the present study, are needed. We emphasize that the bidirectional interpretation of behavioural data is not a new concept in the analysis of rodent behaviour. In the case of other behaviour tests, such as fear conditioning test in rodents, an abnormally short freezing time can be interpreted as a disorder of fear memory, whereas an abnormally long freezing time can be interpreted as a model of post-traumatic stress disorder (PTSD). We believe that such a bidirectional interpretation also can be applied to social behaviours in rodents. Thus, in the present study, if MS mice showed significantly different social behaviour compared to Ctrl mice, we consider it would be an "impairment" beyond the appropriate range of social behaviour, even if the MS mice “stayed closer” than the Ctrl mice.

This inappropriate social distance, which MS mice showed on Day 3 and Day 4, is not found on Days 1 and 2 in both dark and light phases. There is a possibility that at the beginning of the experiment, all the mice were novel to one another, and then the social relationship changed from unfamiliar to familiar after social interaction between cagemates during the experimental period. The results of the reduction of social distance in MS mice emerged on Days 3 and 4, when social relationships likely became more intimate, suggests that MS mice have a different social relationship with "intimate cagemates" compared to Ctrl mice. These results also demonstrate that certain phenotypes in a mouse model can only be detected by conducting long-term behavioural analysis under group-housing conditions.

In our previous report conducted in a similar manner to the present study ^22^, BTBR mice spent less time alone in the novel environment than control C57BL/6 (B6) mice under group-housing conditions. Thereafter, interestingly, we did not observe any significant differences in the amount of time spent alone until Day 4. BTBR mice spent more time alone in the light phase on Day 4. In addition, BTBR mice exhibited a tendency to spend more time with other BTBR mice than with B6 mice during the dark phase, except on Day 1 in the B6-BTBR-mix housing condition. Comparing the results of the previous and present study, we found that the phenotypes of social relationships in mouse models may become more apparent over a longer duration of group housing.

Furthermore, the finding that the phenotype of MS mice in the Ctrl-only and MS-only housing conditions disappeared in the Mix housing condition indicates that the composition of the surrounding members can serve as a risk factor for developing a social disorder in individuals who have been abused in their early-life. In other words, it suggests that proper relationships with others may prevent the onset.

In the Mix housing condition, Ctrl and MS mice showed an inverse correlation in approach preference in the novel environment. This suggests that both Ctrl and MS mice can recognize those that had different early life experiences and express a different preference of approach. One of the possible interpretations of these findings is that there is an experience-driven difference in the behavioural manner of approach during the investigation of a stranger mouse. MS mice prefer to approach those that belong to the same group first, while Ctrl mice prefer to first approach those from a novel group. Another possibility is that MS mice display some form of peculiar behaviour that might draw the attention of the other mice. In contrast to the preference of approach, there was no difference in the total number of approaches between Ctrl and MS mice. However, this finding should be interpreted with caution as mice also exhibit non-social or contingent approaches, and it is difficult to discriminate these approaches from true social approaches in our current analyses because our system only obtains the location of the mouse ID. It might be possible to estimate the number of true social approaches in the future if an improved assay system can obtain not only the location of the mouse ID but also the location of the nose and the base of the tail of the mouse. Thus, such improvements will not only allow for quantitative analysis of the frequency of approach to others but also the qualitative analysis of the manner of approach (speed, angle and contact position). Furthermore, more novel phenotypes of this MS model can be identified by improving assay system in the future.

Additionally, MS mice displayed a lower rank for the time spent on the running wheel than Ctrl mice only on Day 1 under the Mix housing condition. There are two possibilities that explain this phenotype. First, MS mice may have showed an avoidance behaviour against the novel running wheel apparatus. Second, the social rank of MS mice may be lower than Ctrl mice on Day 1. This may be more likely to reflect a novelty response than social rank, as social rank is still considered to be in the process of formation on Day 1. However, in the previous report using IntelliCage, MS mice made more visits to novel corner chambers in the test cage during the first day than controls, and showed competitive subordinance in the water-competition after water-deprivation for 21 h under a group-housed environment^20^. In this previous report, MS mice lost to the control mouse in a water-drinking competition only for the first 5 min, but there was no difference from the control in the total consumption of water during the entire competition period (3 h). Considering this previous research, MS mice may tend to lag in the competition at first. In either case, there is a necessity to be verified with a traditional behaviour test, such as the tube test and novelty response test in the future work.

Sex differences in the effects of early life stress have been reported not only in humans but also in mice^18,28,42–48^. However, in the present study, we investigated the effect of MS on behaviours under group-housing conditions only in male mice, and the effect of MS may be different in female mice. Investigation in both sexes to understand the sex differences in response to early life stress would be important.

In conclusion, we revealed the behavioural phenotypes of MS mice, i.e., inappropriate social distance, alteration of approach preference, and lower rank in the time spent on the running wheel under group-housing conditions. The present findings demonstrate that analyses of animal models under a more ethologically relevant condition, such as a long-term group-housing with social context, should be considered when attempting to understand complex behavioural phenotypes in animal models.

## Methods

### Animals and maternal separation

We prepared mice as previously described^18^. In detail, C57BL/6N female mice at day 14 of pregnancy were purchased from Japan SLC Inc. (Hamamatsu, Japan). Pregnant mice were randomly assigned to Ctrl (n = 20) or MS (n = 21) groups and individually housed on a 12-h light/dark cycle (lights on at 8:00 h), in a temperature-controlled facility (23 °C) with 55% relative humidity. Standard laboratory chow and water were given ad libitum. The date of birth was designated as postnatal day (P) 0. All animal protocols were approved by the Animal Care Committee of Nara Medical University and were performed in accordance with the policies established in the National Institutes of Health Guide for the Care and Use of Laboratory Animals. The study was carried out in compliance with the ARRIVE guidelines. Pups in the MS group were subjected to daily 3 h MS at an unpredictable time during 9:30–18:00 from P 1 to 14. Dams were first removed from their home cages and placed in identical new cages until the end of the separation period. All pups were individually placed in a cup on a heating pad maintained at 32 °C. At the end of the separation period, pups were returned to their home cages, followed by a reunion with their dams. Pups in the Ctrl group were left undisturbed with the dam until weaning, except for cage cleaning once a week. All pups were weaned on P 21 and housed in groups composed of 3–4 of the same gender. The number of male mice used in all experiments is shown in Supplementary Table S1.

### Behavioural analysis under group-housing condition

To quantify behaviour in a group-housed environment, we applied our newly developed assay system. Our assay system is an improved system based on MAPS. Before behavioural experiments, each mouse was tagged with a mouse ID (Fig. 1C) on its back using an elastic string under chloral hydrate anaesthesia (400 mg/kg, intraperitoneally). We take infrared images (10 fps) under infrared illumination and detect precise individual mouse localization in XY coordinates under group-housed conditions. In experiments, a mouse ID occasionally disappears from the image during long-term group-housing (e.g., when mice overlap, or their bodies are tilted). When the system loses the mouse ID, the lost X–Y coordinate is supplemented with previous data from the coordinates where the mouse ID was last identified.

Behavioural analyses began when the male mice were 10–14 weeks old. For the behavioural tests, four adult male mice that had not previously been exposed to one another were placed in an experimental cage (Fig. 1C) from 20:00 on Day 1 and observed continuously for 4 days. In the experiments, three different combinations of mice, i.e., four Ctrl mice (Ctrl-only), four MS mice (MS-only), and two Ctrl mice and two MS mice (Mix), were used (Fig. 1D). We used two cages for the Ctrl-only housing condition, three cages for the MS-only housing condition and six cages for the Mix housing condition, so that in total, Ctrl n = 8 in the Ctrl-only condition, MS n = 12 in the MS-only condition and Ctrl n = 12, MS n = 12 in the Mix condition were used.

### Data analysis

The positioning data from each mouse was exported into comma-separated values (.csv) form. Data processing was carried out using Microsoft R Open 3.5.3 (https://mran.microsoft.com/open).

#### Locomotor activity

Locomotor activity was measured as the total distance travelled (m). The total distance travelled was quantified by 1 h bins or Dark/Light phase (12-h).

#### Inter-individual distances

First, the inter-individual distances between all mouse pairs (6 pairs/cage) were calculated for each frame. The mean inter-individual distances against the other three cagemates' was calculated by averaging the inter-individual distance against the other three mice when focusing on one mouse (focal mouse) during each dark/light phase (12 h) or 1 h bins on each Day. In the case of Mix housing condition, we also calculated the mean inter-individual distances against the other Ctrl and MS mice within a cage (i.e. For MS mice, against MS mouse (MS–MS) was adopted as a distance from another MS mouse in the same experimental cage, while against Ctrl mouse (MS-Ctrl) is calculated as distance from two Ctrl mice).

#### Approach behaviour

The 'social interaction area' was defined for each mouse as a circular area with a 60 mm radius surrounding the mouse ID according to a previous report^21,22^. 'Approach behaviour' was defined as the movement of a mouse into the interaction area of another individual (i.e. the inter-individual distance between two mice was less than 60 mm). The 'approaching mouse' was defined as a mouse moving a longer distance during the last one second (10 frames) when comparing the movements between two mice. 'Total number of approach' for an individual mouse was calculated as the sum of approaches to the other three mice in the same experimental cage every dark/light phase (12 h) or 1 h bins of each day. The 'percentage of approach' was calculated as the ratio; the number of approaches to the same group, i.e. Ctrl mouse to another Ctrl mouse or MS mouse to another MS mouse, was divided by the total number of approaches to all three cagemates. In the Mix housing condition, since two Ctrl mice and two MS mice were in the same cage, the same group mouse other than the subject mouse among the three cagemates was a single mouse. Thus, the chance level in the percentage of approach was 33.3%. 'Total number of approach' and 'percentage of approach' was calculated for 30 min periods in the novel environment (3 h after the start of the experiment) or for dark/light phase (12 h) or 1 h bins on each Day.

#### Time spent on the running wheel

Time spent on the running wheel in the experimental cage for each individual mouse was quantified. The running rank was determined based on the sum of the duration of time during the dark phase for each day, the longer the duration on the running wheel, the higher the ranking.

### Open-field test

Mice were tested for 10 min in an open-field apparatus (39 × 39 × 34 cm). TopScan Lite software (Ver. 2.0, CleverSys Inc., Reston, VA, USA) was used to measure the total distance travelled (m), the time spent in centre (sec) and the number of entries to centre. The centre area was defined as the centre 50% of the apparatus. Mice were used in a separate population from the behavioural test under group-housing condition (Ctrl n = 8, MS n = 7).

### Corticosterone assay

The corticosterone assay was conducted as previously described^49^. Nine-week-old male mice were sacrificed by decapitation, and blood was collected from the trunk side into heparinised tubes. Mice were used in a separate population from the behavioural test (Ctrl n = 7, MS n = 8). Blood was collected during 9:30–12:30 h. Plasma was obtained on centrifugation and stored at − 80 °C until the day of the assay. The concentration of plasma corticosterone was measured using an ELISA kit purchased from Yanaihara Institute Inc. (Hamamatsu, Japan).

### Statistical analysis

For comparing two groups, data were analysed using a Welch two-sample t-test or Mann–Whitney U Test using GraphPad Prism 7.0.4 (GraphPad Software Inc, La Jolla, California, USA). Data with time lapse were tested using a two-way ANOVA (Group × Time), or a three-way ANOVA (Group × Day × Phase or Hour) using ANOVA4 on the Web (Kiriki Kenshi 2002; https://www.hju.ac.jp/~kiriki/anova4). When a significant interaction between factors was observed by ANOVA, a simple main effect test was performed using ANOVA4 on the Web. Statistical significance was set at *p* < 0.05. Graph generation was carried out using GraphPad Prism 7.0.4.

## Supplementary Information

Supplementary Legends.

Supplementary Information 2.

Supplementary Information 3.

## Data Availability

The datasets generated during and/or analysed during the current study are available from the corresponding author on reasonable request.

## Code availability

All scripts used to analyse the data are available upon request.
