# Supplementary material for: The effects of maternal separation on behaviours under social-housing environments in adult male C57BL/6 mice
Source: Sci Rep. 2021 Jan 12;11:527. doi: 10.1038/s41598-020-80206-3 (PMC7804413; doi:10.1038/s41598-020-80206-3)
Supplement: Supplementary file 1 — Supplementary Legends. [file 41598_2020_80206_MOESM1_ESM.docx]

**Supplementary video S1.**

Representative video of behavioural analysis under group-housing environments.
