# Supplementary material for: The effects of maternal separation on behaviours under social-housing environments in adult male C57BL/6 mice
Source: Sci Rep. 2021 Jan 12;11:527. doi: 10.1038/s41598-020-80206-3 (PMC7804413; doi:10.1038/s41598-020-80206-3)
Supplement: Supplementary file 3 — Supplementary Information 3. [file 41598_2020_80206_MOESM3_ESM.pdf]

Supplementary Figures

The effects of maternal separation on behaviours under social-housing environments in adult male C57BL/6 mice

Nozomi Endo[1], Manabu Makinodan[2], Takayo Mannari-Sasagawa[1,3], Noriko Horii-Hayashi[1], Nami Somayama[1], Takashi Komori[2], Toshifumi Kishimoto[2], Mayumi Nishi[1*]

[1]Department of Anatomy and Cell Biology, Nara Medical University, Kashihara 634-8521, Japan
[2]Department of Psychiatry, Nara Medical University, Kashihara 634-8521, Japan
[3]Faculty of Human Life and Environment, Nara Women's University, Nara 630-8506, Japan

[*]Corresponding author:
Mayumi Nishi
Department of Anatomy and Cell Biology, Nara Medical University,
840 Shijo-cho, Kashihara, Nara 634-8521, Japan
e-mail: nmayumi@naramed-u.ac.jp,

Supplementary Table S1: The number of animals used in all experiments.

Mouse Group	Behavioural test under group-housing		Open-field test	Corticosterone assay
	Only-condition	Mix-condition		
Ctrl	8	12	8	7
MS	12	12	7	8

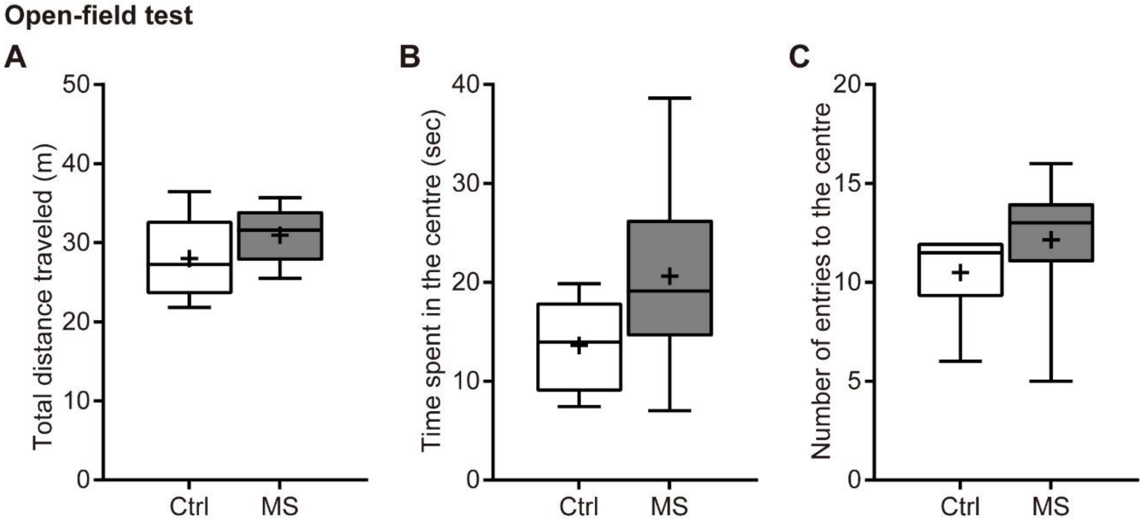

Supplementary Figure S1. The results of the open-field test. (**A**) The total distance travelled (m). (**B**) The time spent in the centre (sec). (**C**) The number of entries to the centre. Centre lines show the medians; box limits indicate the 25th and 75th percentiles; the whiskers show maximum and minimum values; + show the means (Ctrl: n = 8; MS: n =7). Data were analysed using a Welch two-sample t-test.

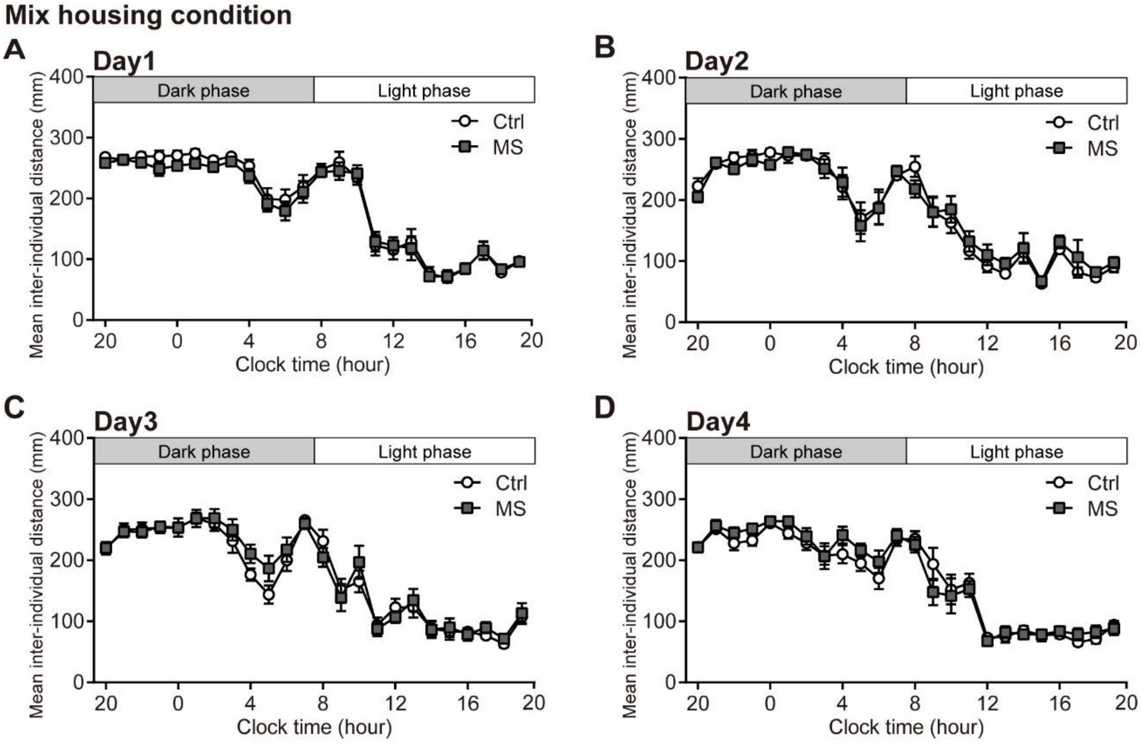

Supplementary Figure S2. The mean inter-individual distances against the other three cagemates under the Mix housing conditions. (**A-D**) The mean inter-individual distances against the other three cagemates divided into 1 hour bins on Days 1–4. Data are shown as the mean ± SE. Data were analysed using three-way ANOVA (Group x Day x Hour). For ease of viewing, the graphs were presented separately for each day. (Ctrl: n = 12; MS: n =12).

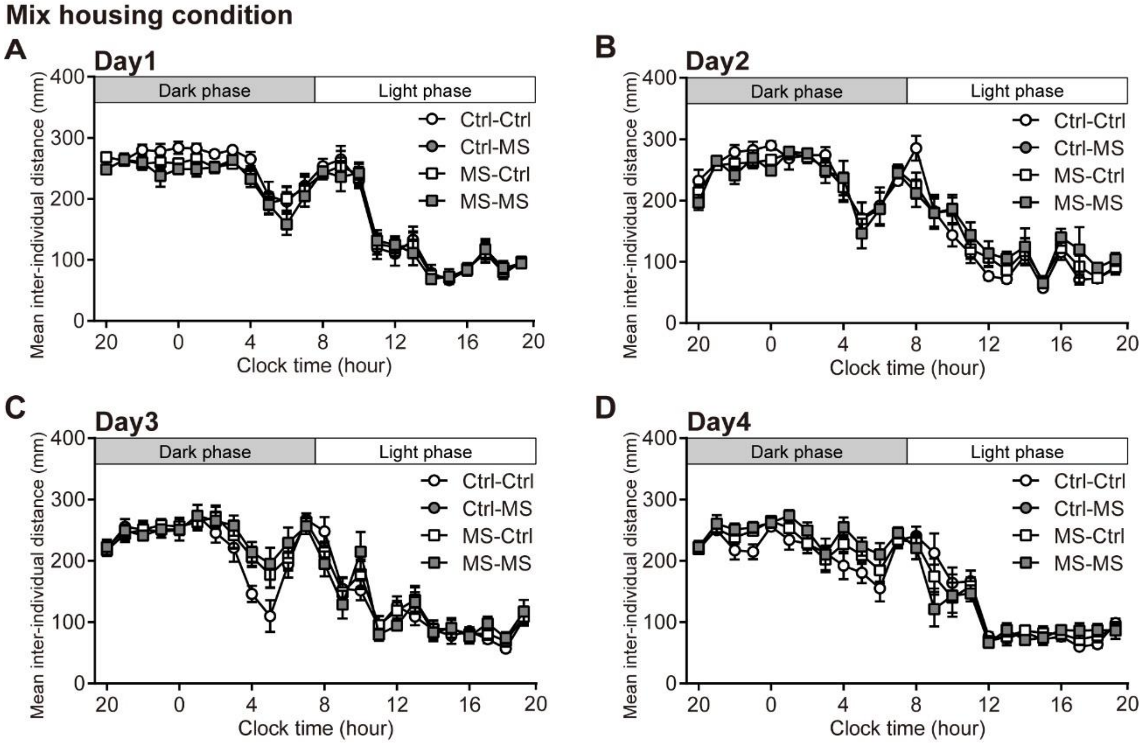

Supplementary Figure S3. The mean inter-individual distances against the other Ctrl mouse and MS mice under the Mix housing conditions. (**A-D**) The mean inter-individual distances against the other Ctrl mouse and MS mice divided into 1 hour bins on Days 1–4. Data are shown as the mean ± SE. Data were analysed using three-way ANOVA (Mice pair x Day x Hour). For ease of viewing, the graphs were presented separately for each day. (n = 12 each).

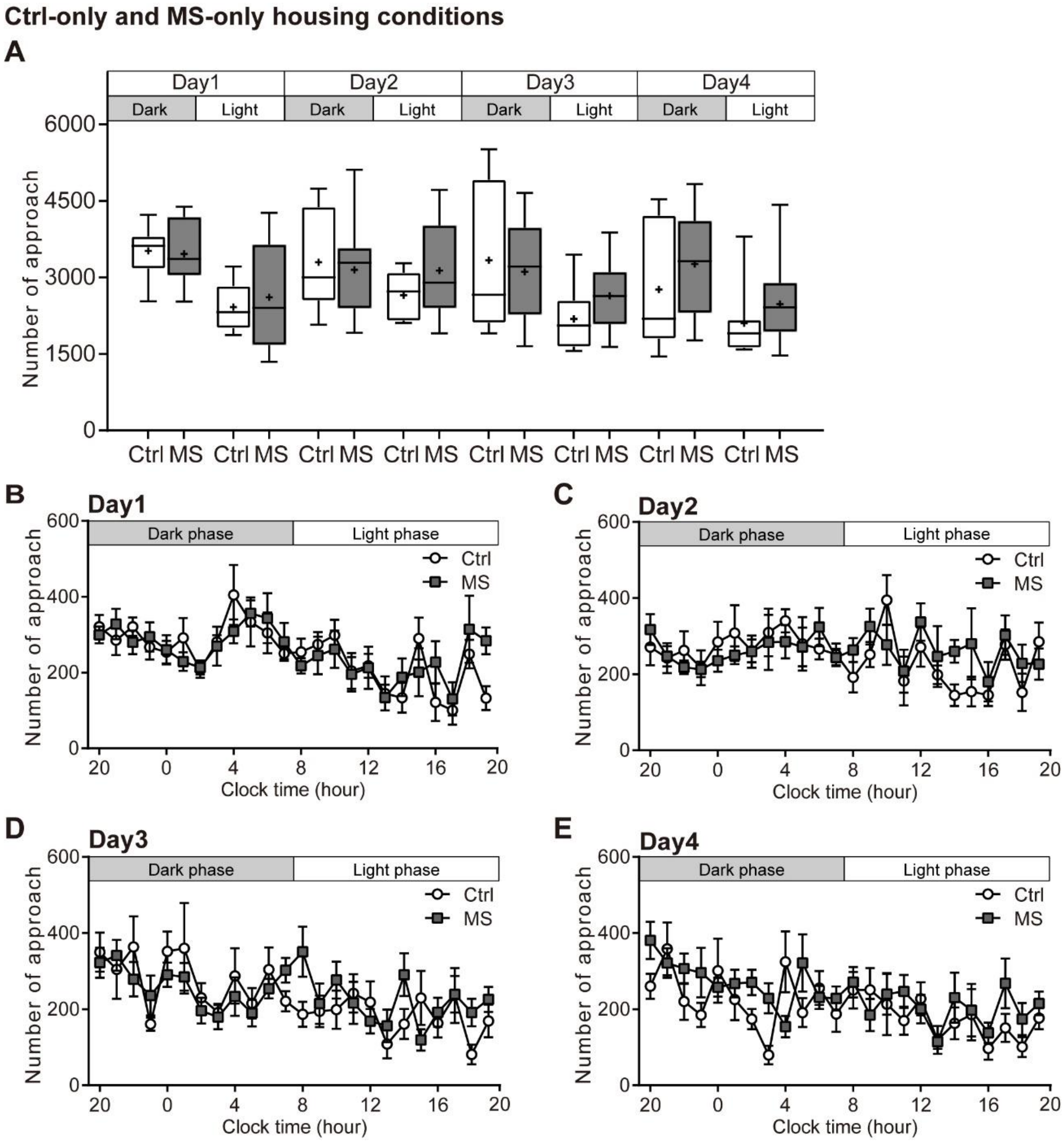

Supplementary Figure S4. The number of approaches under the Ctrl-only and MS-only housing conditions. (**A**) The number of approaches during the dark and light phases (12-h epochs) on Days 1–4. Centre lines show the medians; box limits indicate the 25th and 75th percentiles; the whiskers show maximum and minimum values; + show the means. Data were analysed using three-way ANOVA (Group x Day x Dark/Light phase). (**B-E**) The number of approaches divided into 1 hour bins on Days 1–4. Data are shown as the mean ± SE. Data were analysed using a three-way ANOVA (Group x Day x Hour). For ease of viewing, the graphs were presented separately for each day. (Ctrl: n = 8; MS: n =12).

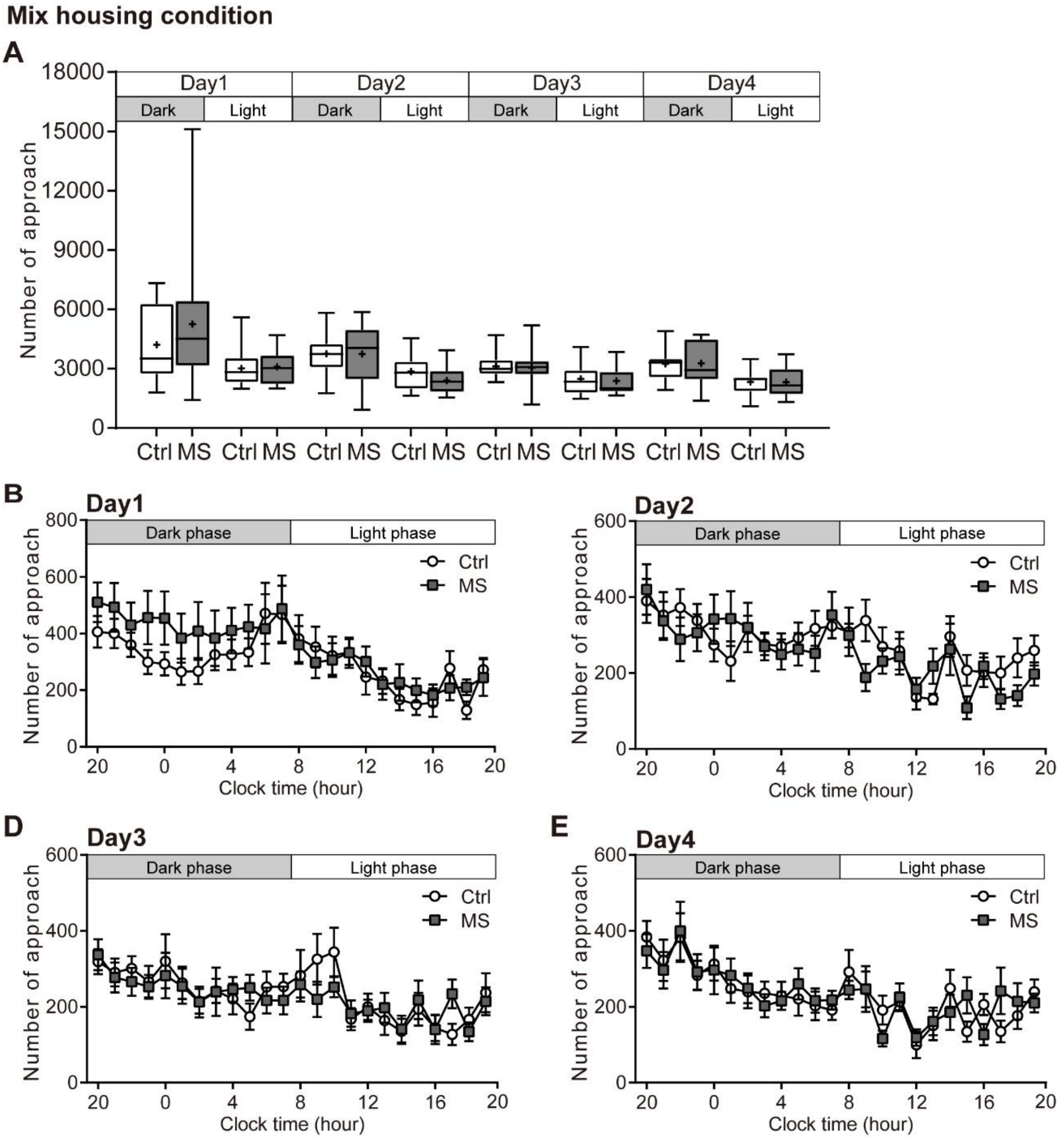

Supplementary Figure S5. The number of approaches under the Mix housing condition. (**A**) The number of approaches during the dark and light phases (12-h epochs) on Days 1–4. Centre lines show the medians; box limits indicate the 25th and 75th percentiles; the whiskers show maximum and minimum values; + show the means. Data were analysed using three-way ANOVA (Group x Day x Dark/Light phase). (**B-E**) The number of approaches divided into 1 hour bins on Days 1–4. Data are shown as the mean ± SE. Data were analysed using a three-way ANOVA (Group x Day x Hour). For ease of viewing, the graphs were presented separately for each day. (Ctrl: n = 12; MS: n =12).

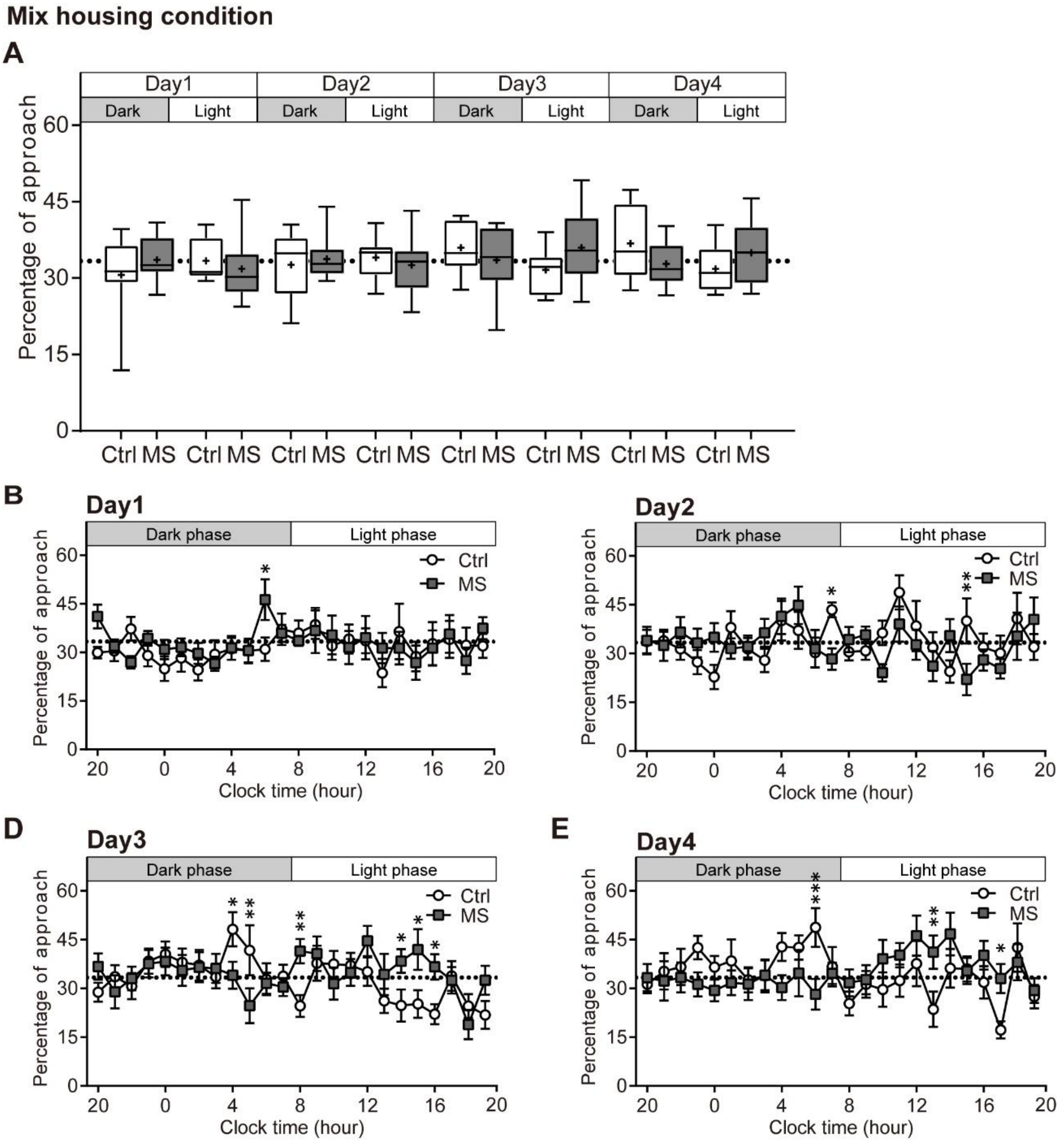

Supplementary Figure S6. The percentage of approaches under the Mix housing condition. (**A**) The percentage of approaches during the dark and light phases (12-h epochs) on Days 1–4. Centre lines show the medians; box limits indicate the 25th and 75th percentiles; the whiskers show maximum and minimum values; + show the means. Data were analysed using a three-way ANOVA (Group x Day x Dark/Light phase). (**B-E**) The percentage of approaches divided into 1 hour bins on Days 1–4. Data are shown as the mean ± SE. Data were analysed using three-way ANOVA (Group x Day x Hour). As there was a significant interaction between factors (p = 0.0006), a simple main effect test was performed. For ease of viewing, the graphs were presented separately for each day. *$p < 0.05$, **$p < 0.01$, ***$p < 0.005$, ****$p < 0.001$. Dash lines indicate chance level. (Ctrl: n = 12; MS: n =12).

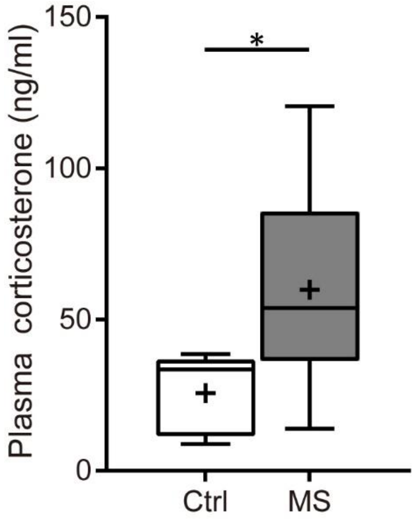

Supplementary Figure S7. Basal plasma corticosterone level. Plasma corticosterone levels of Ctrl and MS mice at 9-weeks old. Centre lines show the medians; box limits indicate the 25th and 75th percentiles; the whiskers show maximum and minimum values; + show the means (Ctrl n = 7, MS n = 8). Data were analysed using a Welch two-sample t-test. *$p < 0.05$ (Ctrl n = 7, MS n = 8). This graph was modified and reprinted from Nishi *et al.*, Folia Pharmacologica Japonica with permission of the publisher[1].

Reference

1. Nishi, M., Sasagawa, T. & Horii-Hayashi, N. Effects of early life adverse experiences on the brain: implications from maternal separation. *Folia Pharmacologica Japonica (Nihon Yakurigaku Zasshi)*. **149**, 72-75 (2017). (in Japanese)
